# Genetic Analysis of ‘*PAX6*-Negative’ Individuals with Aniridia or Gillespie Syndrome

**DOI:** 10.1371/journal.pone.0153757

**Published:** 2016-04-28

**Authors:** Morad Ansari, Jacqueline Rainger, Isabel M. Hanson, Kathleen A. Williamson, Freddie Sharkey, Louise Harewood, Angela Sandilands, Jill Clayton-Smith, Helene Dollfus, Pierre Bitoun, Francoise Meire, Judy Fantes, Brunella Franco, Birgit Lorenz, David S. Taylor, Fiona Stewart, Colin E. Willoughby, Meriel McEntagart, Peng Tee Khaw, Carol Clericuzio, Lionel Van Maldergem, Denise Williams, Ruth Newbury-Ecob, Elias I. Traboulsi, Eduardo D. Silva, Mukhlis M. Madlom, David R. Goudie, Brian W. Fleck, Dagmar Wieczorek, Juergen Kohlhase, Alice D. McTrusty, Carol Gardiner, Christopher Yale, Anthony T. Moore, Isabelle Russell-Eggitt, Lily Islam, Melissa Lees, Philip L. Beales, Stephen J. Tuft, Juan B. Solano, Miranda Splitt, Jens Michael Hertz, Trine E. Prescott, Deborah J. Shears, Ken K. Nischal, Martine Doco-Fenzy, Fabienne Prieur, I. Karen Temple, Katherine L. Lachlan, Giuseppe Damante, Danny A. Morrison, Veronica van Heyningen, David R. FitzPatrick

**Affiliations:** 1 MRC Human Genetics Unit, Institute of Genetics and Molecular Medicine, University of Edinburgh, Western General Hospital, Edinburgh, United Kingdom; 2 Faculty of Medical and Human Sciences, Manchester Centre for Genomic Medicine, Institute of Human Development, University of Manchester, Manchester Academic Health Science Centre (MAHSC), Manchester, United Kingdom; 3 Service de Génétique Médicale, Hôpital de Haute-Pierre, Strasbourg, France; 4 Medical Genetics Departments, University Hospital Jean Verdier, Bondy, France; 5 Department of ophthalmopediatrics, Hôpital Universitaire des Enfants Reine Fabiola, Bruxelles, Belgium; 6 Medical Genetics, Department of Medical Translational Sciences, Federico II University, Naples, Italy; 7 Telethon Institute of Genetics and Medicine (TIGEM), Pozzuoli, Italy; 8 Department of Ophthalmology, Justus-Liebig-University Giessen, Universitaetsklinikum Giessen and Marburg UKGM, Giessen, Germany; 9 Institute of Child Health, University College London, UK and Great Ormond Street Hospital for Children, London, United Kingdom; 10 Northern Ireland Regional Genetics Service (NIRGS), Belfast City Hospital, Belfast, United Kingdom; 11 Department of Eye and Vision Science, Institute of Ageing and Chronic Disease, University of Liverpool, Liverpool, United Kingdom; 12 Medical Genetics Unit, St George's University of London, London, United Kingdom; 13 Moorfields Eye Hospital, London, UK and University College London, Institute of Ophthalmology, London, United Kingdom; 14 Department of Pediatric Genetics, University of New Mexico Health Sciences Center, Albuquerque, New Mexico, United States of America; 15 Centre de Génétique Humaine, Université de Franche-Comté, Besançon, France; 16 Clinical Genetics Unit, Birmingham Women's Hospital, Birmingham, United Kingdom; 17 Department of Clinical Genetics, University Hospitals, Bristol, United Kingdom; 18 Center for Genetic Eye Diseases, Cole Eye Institute, Cleveland Clinic Foundation, Cleveland, OH, United States of America; 19 Department Ophthalmology, University Hospital of Coimbra, Coimbra, Portugal; 20 Children's Hospital, Doncaster Royal Infirmary, Doncaster, United Kingdom; 21 Human Genetics Unit, University of Dundee College of Medicine, Dentistry and Nursing, Ninewells Hospital, Dundee, United Kingdom; 22 Department of Ophthalmology, Princess Alexandra Eye Pavilion, Chalmers Street, Edinburgh, United Kingdom; 23 Institut für Humangenetik, Universitätsklinikum Essen, Universität Duisburg-Essen, Essen, Germany; 24 Institut für Humangenetik, Universitätsklinikum Düsseldorf, Heinrich-Heine-Universität Düsseldorf, Düsseldorf, Germany; 25 Center for Human Genetics, Freiburg, Germany; 26 Department of Life Sciences, Glasgow Caledonian University, Glasgow, United Kingdom; 27 Clinical Genetics, Southern General Hospital, Glasgow, United Kingdom; 28 Department of Paediatrics and Child Health, Ipswich Hospital, Ipswich, United Kingdom; 29 North East Thames Regional Genetics Service, Great Ormond Street Hospital for Children NHS Foundation Trust, Great Ormond Street Hospital, London, United Kingdom; 30 Ruber International Hospital, Medical Genetics Unit, Mirasierra, Madrid, Spain; 31 Northern Genetics Service, Institute of Genetic Medicine, Newcastle upon Tyne Hospitals NHS Foundation Trust, Newcastle Upon Tyne, United Kingdom; 32 Department of Clinical Genetics, Odense University Hospital, Odense C, Denmark; 33 Department of Medical Genetics, Oslo University Hospital, Oslo, Norway; 34 Department of Clinical Genetics, Churchill Hospital, Oxford University Hospitals NHS Trust, Oxford, United Kingdom; 35 UPMC Eye Center, Children's Hospital of Pittsburgh of UPMC, School of Medicine, University of Pittsburgh, Pittsburgh, Pennsylvania, United States of America; 36 Service de génétique, HMB CHU Reims, SFR Cap Sante. EA 3801, France; 37 CHU de Saint Etienne, Service de génétique médicale, Saint-Etienne, France; 38 Academic Unit of Genetic Medicine, Division of Human Genetics, University of Southampton, Southampton, United Kingdom; 39 Wessex Clinical Genetics Service, University Hospital Southampton NHS Foundation Trust, Southampton, United Kingdom; 40 Department of Medical and Biological Sciences, University of Udine, Udine, Italy; 41 St. Thomas’ Hospital, Westminster Bridge Road, London, United Kingdom; University of Iowa, UNITED STATES

## Abstract

We report molecular genetic analysis of 42 affected individuals referred with a diagnosis of aniridia who previously screened as negative for intragenic *PAX6* mutations. Of these 42, the diagnoses were 31 individuals with aniridia and 11 individuals referred with a diagnosis of Gillespie syndrome (iris hypoplasia, ataxia and mild to moderate developmental delay). Array-based comparative genomic hybridization identified six whole gene deletions: four encompassing *PAX6* and two encompassing *FOXC1*. Six deletions with plausible *cis*-regulatory effects were identified: five that were 3ʹ (telomeric) to *PAX6* and one within a gene desert 5ʹ (telomeric) to *PITX2*. Sequence analysis of the *FOXC1* and *PITX2* coding regions identified two plausibly pathogenic *de novo FOXC1* missense mutations (p.Pro79Thr and p.Leu101Pro). No intragenic mutations were detected in *PITX2*. FISH mapping in an individual with Gillespie-like syndrome with an apparently balanced X;11 reciprocal translocation revealed disruption of a gene at each breakpoint: *ARHGAP6* on the X chromosome and *PHF21A* on chromosome 11. In the other individuals with Gillespie syndrome no mutations were identified in either of these genes, or in *HCCS* which lies close to the Xp breakpoint. Disruption of *PHF21A* has previously been implicated in the causation of intellectual disability (but not aniridia). Plausibly causative mutations were identified in 15 out of 42 individuals (12/32 aniridia; 3/11 Gillespie syndrome). Fourteen of these mutations presented in the known aniridia genes; *PAX6*, *FOXC1* and *PITX2*. The large number of individuals in the cohort with no mutation identified suggests greater locus heterogeneity may exist in both isolated and syndromic aniridia than was previously appreciated.

## Introduction

Abnormal development of the iris is a feature of a variety of congenital human ocular anomalies, of which, the best characterized is complete aniridia (MIM 106210), a dominantly inherited condition with an incidence of less than 1 in 50,000 [1]. Aniridia presents as congenital absence of the iris, although a visible partial rim or sector of iris tissue strand is often present [2]. Foveal hypoplasia, cataract, keratopathy and glaucoma sometimes develop in second or third decade contributing to visual morbidity [3]. Non-ocular anomalies including hyposmia and structural brain changes are sometimes observed in individuals with complete aniridia [4].

At least 90% of aniridia cases are caused by heterozygous loss-of-function mutations in *PAX6* [5]. Almost all cases of classical aniridia associated with *PAX6* haploinsufficiency present with foveal hypoplasia. Heterozygous, presumed hypomorphic, missense mutations in *PAX6* have also been associated with other ocular diseases including anterior segment dysgenesis [6] and optic nerve malformations [7]. Rarely, isolated aniridia is caused by mutations in *FOXC1* [8,9] or *PITX2* [10]. Mutations in these genes are more commonly associated with juvenile-onset glaucoma [11] and anterior segment dysgenesis [12–14] presenting with syndromic features of rare cardiac anomalies for *FOXC1* and hypodontia and umbilical anomalies for *PITX2*.

Several syndromic forms of iris developmental anomalies have been described. The best known is WAGR (Wilms’ tumour, aniridia, genital anomalies and mental retardation; MIM 194072), a contiguous deletion syndrome on 11p13 [15]. Gillespie syndrome (MIM 206700) is characterized by a pathognomonic iris anomaly; absence of the pars pupillaris of the iris and the pupillary border. Individuals with Gillespie syndrome are also distinguished from complete aniridia by having a normal fovea and no evidence of progressive opacification of the cornea and lens, nor development of glaucoma. The extraocular features are non-progressive cerebellar ataxia and psychomotor delay [16]. Several cases of Gillespie syndrome have been reported [17–39].

In the literature, Gillespie syndrome has been most commonly considered to be an autosomal recessive disorder [36–38]. Analysis of the *PAX6* gene in six Gillespie syndrome patients revealed no intragenic mutations [20,26,40]. *PAX6* mutations have been reported in two individuals [33,39] described as Gillespie syndrome but with significantly atypical features such as corectopia and ptosis (33). A single affected girl described as having a Gillespie syndrome-like phenotype has been reported with an apparently balanced X:autosome reciprocal translocation t(X;11)(p22.32;p12) [22] and atypical features of superior coloboma, foveal hypoplasia and vermis hypoplasia. This case is included in this study as individual 1371.

Here, we report genomic copy number and extended mutation analysis in 42 unrelated affected individuals all of whom had been scored as negative for intragenic *PAX6* mutations. Eleven of these probands had been referred to us with a diagnosis of Gillespie syndrome and 31 with non-syndromic aniridia. One of the 11 Gillespie syndrome individuals was the case with the apparently balanced reciprocal translocation t(X:11)(p22.32;p12) [22]. In this case we used FISH to map both breakpoints. In total, 15 plausible disease-causing heterozygous loss-of-function mutations were identified: nine affecting *PAX6*, four affecting *FOXC1*, one affecting *PITX2* and one affecting *PHF21A*. These data suggest that other disease loci or mutational mechanisms causing aniridia remain to be discovered.

## Materials and Methods

### Patient samples

All aspects of this study were performed in accordance with the Declaration of Helsinki. Written informed consent was obtained from the participants and recorded. The study was approved by the UK Multicentre Regional Ethics Committee under the number 06/MRE00/76. All patients were phenotypically characterized by experienced ophthalmologists or geneticists. The study cohort consisted of 42 unrelated individuals with aniridia or Gillespie syndrome (S1 Table) each of whom had been previously screened for intragenic *PAX6* mutations by single-strand conformation polymorphism (SSCP), denaturing high performance liquid chromatography (DHPLC) and direct sequencing (S2 Table).

### DNA preparation and quality control

Genomic DNA was prepared from either lymphoblastoid cell lines (LCL) or saliva using a Nucleon DNA extraction kit (Tepnel Life Sciences, UK). DNA quality was checked by agarose gel electrophoresis and NanoDrop spectrophotometry (Thermo Scientific).

### Array comparative genomic hybridization (aCGH)

Genome-wide analysis of DNA copy number was carried out using the Roche Nimblegen 12X135k whole-genome array (median probe spacing of approximately 12 kb) according to the manufacturer’s instructions with minor modifications, as described previously [41].

Targeted analysis of genomic deletions/duplications was performed using a customized oligonucleotide microarray (Agilent Technologies) consisting of 44,000 60-mer oligonucleotide probes (4X44k), designed using eArray (Agilent Technologies). The design consisted of a 3 Mb genomic region (chr11:30,262,916–33,296,085; hg18) containing the *PAX6* gene with an average probe spacing of 76 bp. ‘Dye-swap’ experiments were performed followed by copy number analysis, as previously described [42].

### Polymerase chain reaction (PCR) and mutation analysis

Primer sequences and PCR conditions used for amplification and sequencing of the *FOXC1*, *PITX2*, *PHF21A* and *ARHGAP6* genes are provided in S2 Table. PCR reactions were performed in 12μl volumes containing 1μl of 1-in-20 diluted, whole-genome amplified DNA (Genomiphi, GE Healthcare), 6μl of 2 X ReddyMix PCR Mastermix (Abgene), 833 nM of each oligonucleotide primer and 2.4μl of 5 X GC-mix (where appropriate). PCR conditions generally consisted of an initial denaturation at 95°C for 5 minutes, followed by 32 cycles of 94°C for 60 seconds, primer annealing for 60 seconds, and 72°C for 60 seconds, and a final cycle of 72°C for 10 minutes. The products were visualized using agarose gel electrophoresis to ensure adequate yield and proper sizing of each exon fragment. Sequencing of PCR products was performed in both directions as described elsewhere [43]. Sequence traces were analyzed using Mutation Surveyor sequence analysis software version 3.30.

### Fluorescence *in situ* hybridization (FISH)

Metaphase spreads for FISH were prepared from patient lymphocytes as described elsewhere [44]. BAC clones were selected from the Ensembl database (http://www.ensembl.org) or the UCSC Human Genome Browser (http://genome.ucsc.edu) and ordered from the BACPAC resources centre (Children’s Hospital Oakland Institute). For the initial mapping of the clones, DNA was isolated using a rapid alkaline lysis miniprep method (Qiagen mini/midi plasmid kit). Probes were labeled with biotin-16-dUTP or digoxigenin-11-dUTP (Roche) by nick translation. Probe labelling, DNA hybridization and antibody detection were carried out as described previously [45]. Following hybridization, slides were mounted with a drop of Vectorshield antifadent containing DAPI (Sigma). Antibody detection was carried out by fluorescent microscopy using a Zeiss Axioscop microscope. Images were collected using a cooled CCD (charged coupled device) camera and analyzed using SmartCapture software (Digital Scientific).

## Results

### Patient cohort

Our study cohort consisted of 42 unrelated individuals (14 male, 28 female) with iris developmental anomalies (Table 1, S1 Table). Eleven of these individuals (2 male, 9 female) had been referred to us with a diagnosis of Gillespie syndrome including individual 1371 who had been previously reported with an apparently balanced reciprocal translocation: t(X;11)(p22.32;p12) [22]. Each proband had been scored negative for intragenic *PAX6* mutations by SSCP, DHPLC and/or direct sequencing in our lab.

**Table 1:** Details of the clinical diagnoses and genetic pathology identified in individuals in this study.

Individual ID	DECIPHER ID	Clinical feature	Genetic pathology	Genomic coordinates (hg18)
1851 (control)	323119	Aniridia	*PAX6* deletion (previously identified by FISH)	chr11:21,254,000–32,564,000
2193	323118	Aniridia	*PAX6* whole-gene deletion	chr11:31,199,000–31,849,000
377	323104	Aniridia	*PAX6* whole-gene deletion	chr11:31,394,000–31,914,000
1510	323113	Aniridia	*PAX6* whole-gene deletion	chr11:31,779,000–31,933,000)
1977	323116	Aniridia	*PAX6* whole-gene deletion	chr11:31,698,271–31,794,414
1514	323114	Aniridia	*PAX6* telomeric deletion	chr11:30,874,642–31,654,833
753	323108	Aniridia	*PAX6* telomeric deletion	chr11:30,967,000–31,704,000)
555	323106	Aniridia	*PAX6* telomeric deletion	chr11:31,108,579–31,649,842)
2014	323117	Gillespie syndrome	*PAX6* telomeric deletion	chr11:31,234,395–31,751,815
659	323107	Aniridia	*PAX6* telomeric deletion	chr11:31,379,000–31,708,000)
1449	323112	Gillespie syndrome	*FOXC1* whole-gene deletion	chr6:1,543,591–1,675,085
1246	323110	Aniridia	*FOXC1* whole-gene deletion	chr6:1,543,591–1,675,085
1839		Aniridia	*FOXC1* c.235C>A p.(Pro79Thr) *de novo*	Not applicable
1634		Aniridia	*FOXC1* c.302T>C p.(Leu101Pro) *de novo*	Not applicable
1194	323109	Aniridia	*PITX2* telomeric deletion	chr4:111,994,000–115,504,000
1371	n/a	Gillespie syndrome	Translocation t(X;11)(p22.32;p12)	See Fig 5

### DNA copy number analysis of the *PAX6* locus

**Fig 1:**
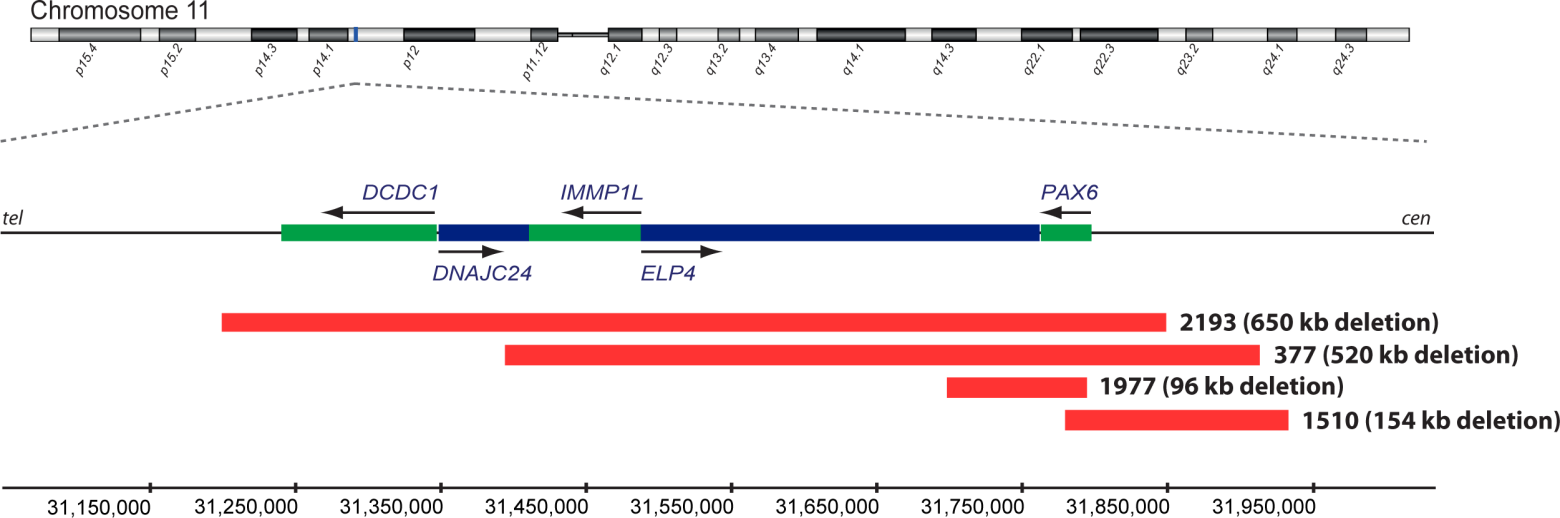
Identification of *PAX6* whole-gene deletions. Genome-wide array CGH analysis identified a 650 kb deletion in individual 2193 (chr11:31,199,000–31,849,000), a 520 kb deletion in individual 377 (chr11:31,394,000–31,914,000), a 154 kb deletion in individual 1510 (chr11:31,779,000–31,933,000) and a 96 kb deletion in individual 1977 (chr11:31,698,271–31,794,414), all involving *PAX6*. Red bars show the position of the deletions. Genes transcribed on the forward strand are in blue and those transcribed on the reverse strand are in green, also indicated by arrows. Genomic coordinates are based on the Human Genome Assembly hg18.

**Fig 2:**
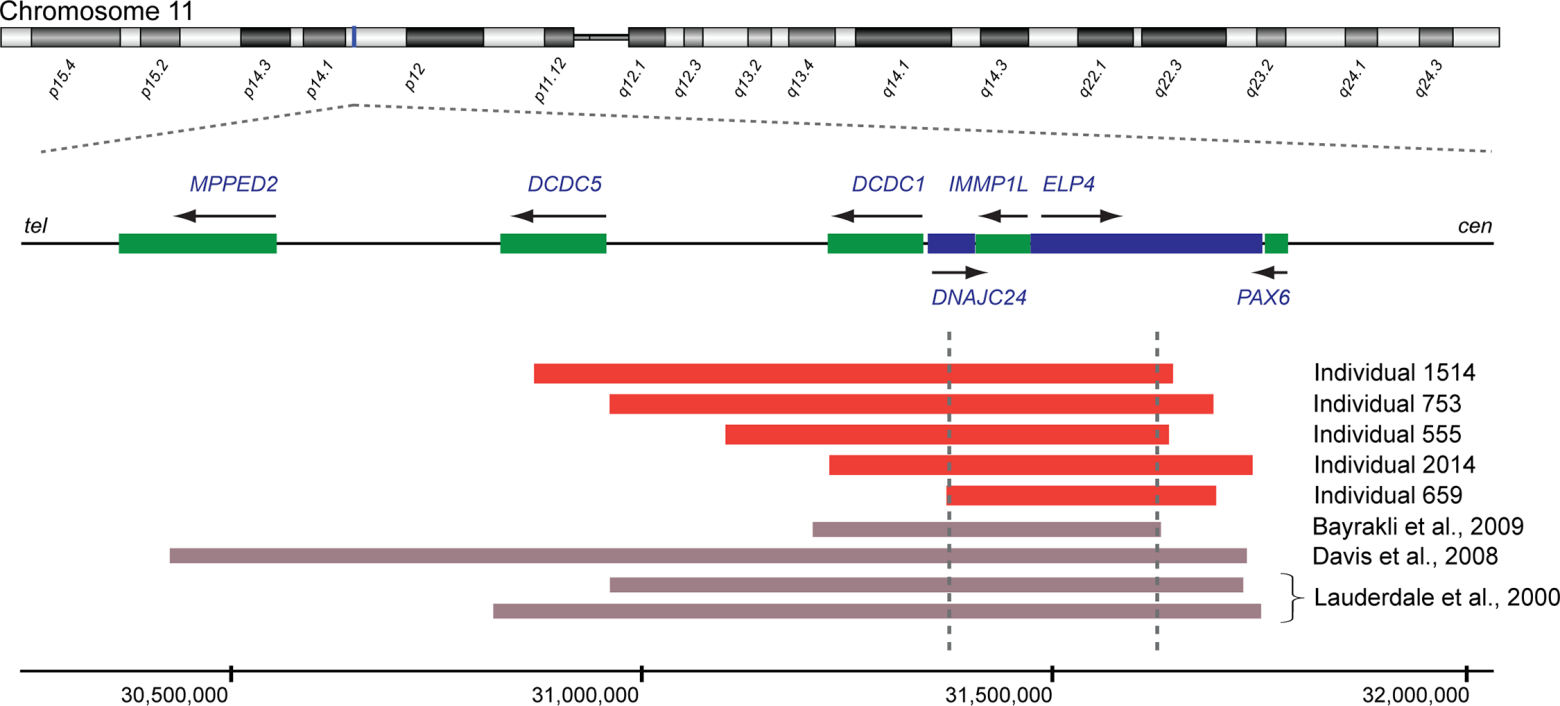
Identification of regulatory deletions telomeric to *PAX6*. Regulatory deletions telomeric to *PAX6* were identified in individual 1514 (chr11:30,874,642–31,654,833), individual 753 (chr11:30,967,000–31,704,000), individual 555 (chr11:31,108,579–31,649–842), individual 2014 (chr11:31,234,395–31,751,815) and individual 659 (chr11:31,379,000–31,708,000). The schematic diagram shows how the ‘critical region’ (delimited by grey dotted lines) required for *PAX6* transcriptional activation was delineated by combining our data with published deletions with known coordinates [55,67,68]. *PAX6* regulatory deletions from the present study are shown by red blocks. Genes transcribed on the forward strand are in blue and those transcribed on the reverse strand are in green, also indicated by arrows. Genomic coordinates are based on the Human Genome Assembly hg18.

To identify causative segmental aneuploidy, two array-based comparative genomic hybridization (aCGH) approaches were used: a 135k whole-genome array and a custom-designed targeted array covering a contiguous 3 Mb genomic region (chr11:30,262,916–33,296,085; hg18) encompassing *PAX6*. This identified four individuals with heterozygous deletions, all encompassing *PAX6* and ranging in size from 96 kb to 650 kb: individual 2193 (chr11:31,199,000–31,849,000; hg18), individual 377 (chr11:31,394,000–31,914,000; hg18), individual 1510 (chr11:31,779,000–31,933,000; hg18) and individual 1977 (chr11:31,698,271–31,794,414; hg18) (Table 1, Fig 1, S1 Fig). Five individuals had deletions with breakpoints immediately telomeric to *PAX6*: individual 1514 (chr11:30,874,642–31,654,833; hg18), individual 753 (chr11:30,967,000–31,704,000; hg18), individual 555 (chr11:31,108,579–31,649–842; hg18), individual 2014 (chr11:31,234,395–31,751,815; hg18) and individual 659 (chr11:31,379,000–31,708,000; hg18) (Table 1, Fig 2, S2 Fig). Combining these with published data, we suggested a 243.9 kb critical region for *PAX6* transcriptional activation between chr11:31,379,000 (hg18) and chr11:31,622,916 (hg18) (Fig 2).

### Mutation analysis of the *FOXC1* locus

**Fig 3:**
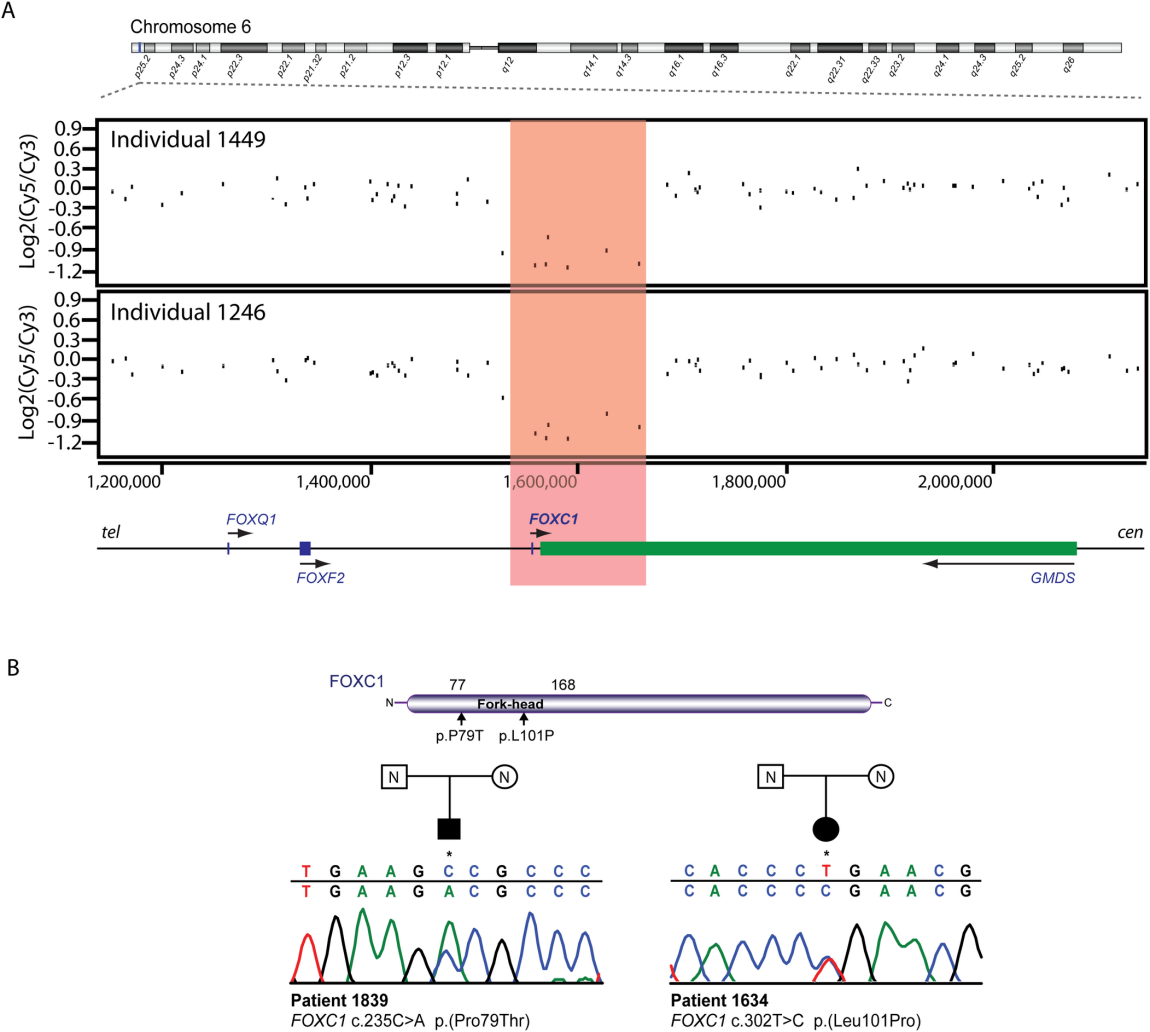
Mutation analysis of the *FOXC1* locus. **(A)** Genome-wide array CGH identified two deletions encompassing the *FOXC1* gene in individuals 1449 (chr6:1,543,591–1,675,085) and 1246 (chr6:1,543,591–1,675,085). **(B)** Direct sequencing of the *FOXC1* coding region identified a heterozygous substitution in individual 1839 (c.235C>A, p.(Pro79Thr)) and another in individual 1634 (c.302T>C, p.(Leu101Pro)). *FOXC1* mutation screening in unaffected parents of both patients showed that the mutations had occurred *de novo*. The locations of both mutations within the fork-head domain of the FOXC1 protein are indicated by vertical arrows. Genes transcribed on the forward strand are in blue and those transcribed on the reverse strand are in green, also indicated by arrows. Genomic coordinates are based on the Human Genome Assembly hg18. The genomic sequence identifier for *FOXC1* is NG_009368.

An apparently identical 131 kb deletion (chr6:1,543,591–1,675,085; hg18) encompassing *FOXC1* was identified as a *de novo* occurrence in two unrelated individuals 1449 and 1246 (Table 1, Fig 3). Each of these deletions had been confirmed in an independent UK laboratory using an alternative method. Furthermore, the two individuals were shown to be distinct based on their aCGH profile of genome-wide copy number variants (data not shown). We then screened *FOXC1* in our cohort by direct sequencing. Two individuals were found to carry missense mutations in the *FOXC1* fork-head domain (Table 1, Fig 3). Individual 1839 had a C>A transversion in codon 79 (c.235C>A, p.(Pro79Thr)) and individual 1634 had a novel T>C transition in codon 101 (c.302T>C, p.(Leu101Pro)). In both individuals, the mutations were absent from the unaffected parents and had most likely occurred *de novo* (Fig 3). The amino acid substitution p.(Pro79Thr) has been reported previously in a family with classical Axenfeld-Rieger syndrome and the mutant protein has impaired nuclear localization and transactivation activity [46]. The novel p.(Leu101Pro) mutation is predicted to disrupt the second alpha helix of the fork-head domain.

### Mutation analysis of the *PITX2* locus

**Fig 4:**
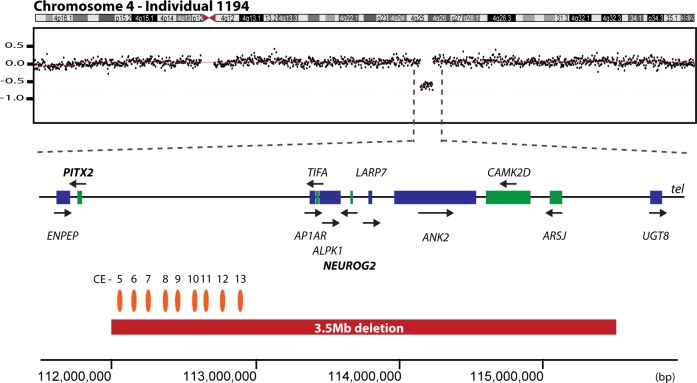
Identification of a potential *PITX2* regulatory deletion. Genome-wide array CGH identified a deletion of approximately 3.5 Mb in individual 1194 (chr4:111,994,000–115,504,000) (red bar). The deletion is located telomeric to the *PITX2* gene on chromosome 4. The positions of conserved elements (CE) in the deleted region, as identified by Volkmann et al., 2011 [47] are marked by orange ellipses. Genes transcribed on the forward strand are in blue and those transcribed on the reverse strand are in green, also indicated by arrows. Genomic coordinates are shown on the x-axis and are based on the Human Genome Assembly hg18.

Array CGH identified a 3.5 Mb deletion of 4q25-q26 (chr4:111,994,000–115,504,000; hg18) in individual 1194 (Table 1, Fig 4). This deletion encompasses 8 genes (Fig 4). The centromeric breakpoint is located in a gene desert 230 kb telomeric (5ʹ) to *PITX2* encompassing several conserved *PITX2* enhancer elements [47]. Subsequent screening of the *PITX2* coding sequence in our cohort revealed no plausible disease-causing mutations.

### Breakpoint mapping of a translocation in an individual with Gillespie syndrome

**Fig 5:**
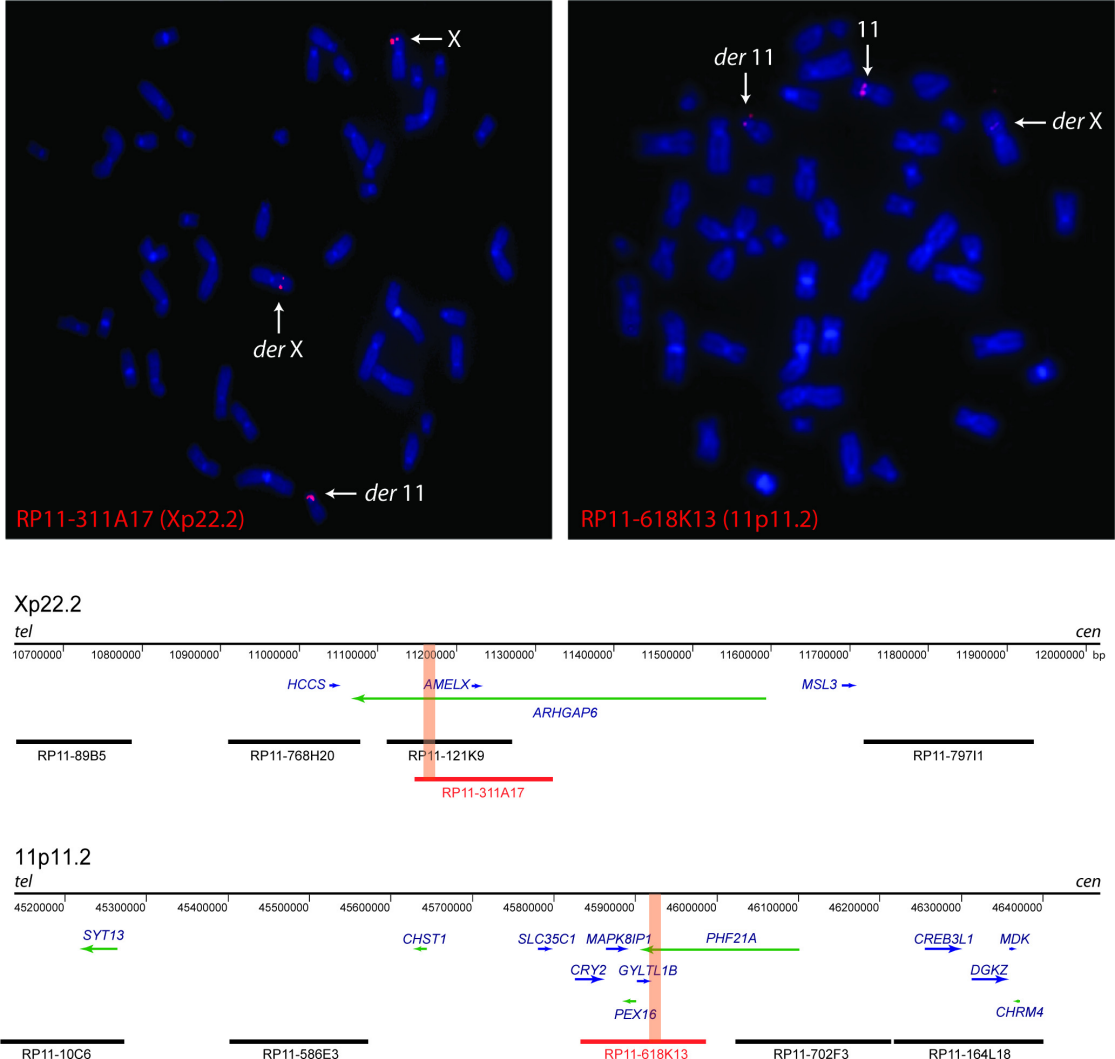
Fluorescence *in situ* hybridization (FISH) was used to map the translocation breakpoints on chromosomes 11 and X in individual 1371. The breakpoint-spanning BAC clones RP11-311A17 (Xp22.2; left panel) and RP11-618K13 (11p11.2; right panel) show signals on both the derivative 11 and derivative X. The schematic diagram demonstrates the position of the BAC clones and the genes involved, to scale. Breakpoint-spanning BACs are coloured in red, with the approximate position of the breakpoints shown by orange bars, as determined by long-range PCR. Genes transcribed on the forward strand are in blue and those transcribed on the reverse strand are in green. Genomic coordinates are shown on the x-axis and are based on the Human Genome Assembly hg18.

FISH was used to map the previously reported t(X;11)(p22.32;p12) reciprocal translocation in individual 1371 (Fig 5). The breakpoint on chromosome 11 (now 11p11.2) lay within a single BAC, RP11-618K13 [48], which contains 5 known genes, *CRY2*, *MAPK8IP1*, *PEX16*, *GYLTL1B*, and *PHF21A* (also known as *BHC80*), located approximately 14.1 Mb centromeric to *PAX6*. The breakpoint was shown to lie within *PHF21A* using probes generated by long-range PCR from exons 14–16 (telomeric to the breakpoint) and exons 4–11 (centromeric to the breakpoint) (data not shown). The X chromosome breakpoint (now Xp22.2) was spanned by two overlapping BACs (RP11-121K9 and RP11-311A17) [48] covering two genes, *AMELX* and *ARHGAP6* (Fig 5). Using a probe generated by long-range PCR, the breakpoint was localized within a large intron of *ARHGAP6* (Fig 5).

### Mutation analysis of breakpoint genes in Gillespie syndrome patients

Direct sequencing of the coding exons and essential splice sites of *PHF21A* and *ARHGAP6* revealed only polymorphic variants in the 10 individuals with Gillespie syndrome who lacked a detectable chromosomal abnormality at these loci. *HCCS* is located approximately 150 kb telomeric to the X chromosome breakpoint in individual 1371. Mutations in this gene have been associated with microphthalmia with linear skin defects (MIM 309801). Direct sequencing of *HCCS* revealed no mutations in the 10 non-translocation Gillespie cases.

## Discussion

A high proportion of cases of aniridia is caused by loss-of-function mutations in a single gene, *PAX6*. Here we studied individuals with aniridia and Gillespie syndrome, who had previously scored negative for intragenic *PAX6* mutations, using a variety of molecular approaches to identify causative mutations. The rationale for the analysis was that we had a strong prior expectation that this cohort would be heavily enriched for causative structural chromosomal anomalies involving *PAX6* itself, but also for possible new disease loci and/or novel mutational mechanisms. In the event, we identified deletions that result in *PAX6* haploinsufficiency in only 9/42 probands: four encompassing *PAX6* itself and five removing 3ʹ (telomeric) *cis*-regulatory elements that are essential for PAX6 function. A wealth of evidence exists from animal models [49–52] and human translocation breakpoint mapping [53,54] showing that genomic elements located in a region ~120kb 3ʹ to the transcription unit are essential for the transcriptional activation of *PAX6*. For chromosomal deletions the most convincing evidence is from somatic cell hybrid analysis of two deletions that were shown to abolish *PAX6* transcription [55]. The deletions studied in this somatic cell hybrid analysis both overlap with the 3ʹ deletions identified here (Fig 2) and by combining our data with the published data we suggest a new 244 kb ‘critical region’ which contains essential *cis*-regulatory elements (Fig 2). The patient cohort in the present study is part of a larger cohort of iris developmental anomalies patients in which one individual with aniridia was recently found to have a plausibly causative *de novo* single nucleotide variant (SNV) in a conserved non-coding element within the ‘critical region’ [56]. While it is possible that similar mutations may exist in other *cis*-regulatory elements, it is significant that most of the individuals in the present study were included in the cohort of 60 individuals screened for *PAX6* regulatory mutations by Bhatia *et al*. [56] and no further mutations were identified in the regions analyzed.

Four individuals had deletions or intragenic mutations which are likely to result in *FOXC1* haploinsufficiency. One individual had a large deletion upstream of *PITX2* that plausibly impairs developmental expression of this gene by removing known enhancer elements. Deletions of *FOXC1* were previously shown to account for a considerable proportion of individuals with anterior segment dysgenesis, who also presented with extraocular features such as hearing defects and mental retardation [57]. *FOXC1* and *PITX2* encode transcriptional regulators that physically interact with each other and are co-expressed in a number of tissues during development including the periocular mesenchyme [58]. Mutations in these genes have most commonly been associated with Axenfeld-Rieger syndrome [59], but aniridia has been reported for both [8,9,60]. Of note, 3 of the 4 individuals reported here with *FOXC1* haploinsufficiency, and the individual with the *PITX2 cis*-regulatory mutation have congenital glaucoma associated with their aniridia phenotype. However, none of the nine individuals with *PAX6* mutations had congenital glaucoma. Digenic inheritance of *FOXC1* and *PITX2* mutations was reported in a severely affected individual in a family with several affected members presenting with variable ocular phenotypes associated with Axenfeld-Rieger syndrome [13]. The presence of both FOXC1 and PITX2 mutations impaired the transactivation activity of these proteins *in vitro* significantly more than when only one mutation was present. The cellular and developmental interactions between PAX6, FOXC1 and PITX2, and physical co-binding at regulatory elements in the developing iris are as yet poorly understood. This is presumably due to the difficulty in obtaining sufficient tissue, although the available human genetic data suggest that this would be an informative area of study.

We assessed the occurrence of particular descriptive phenotype terms (partial/variant aniridia, corneal anomalies, cataracts, glaucoma, microphthalmia/coloboma and extraocular features) in cases with and without a molecular diagnosis (S3 Table). The results showed an over-representation of individuals with partial/variant aniridia in whom no genetic defect was detected (approximately 60%) when compared to those with the same descriptive term but no genetic diagnosis (approximately 26%). This finding can be explained by the presence of 8/11 Gillespie syndrome patients in whom a genetic mutation is yet to be identified. The glaucoma feature appeared to be present in 26% of individuals with a molecular diagnosis (particularly in *FOXC1* and *PITX2* mutation-positive patients) compared to 7% of those without a diagnosis.

Finally, we report a more complex mutation associated with the breakpoints of a balanced X:autosome translocation in a single individual. On chromosome 11 the breakpoint disrupts *PHF21A*, which encodes a plant-homeodomain zinc finger protein and is highly expressed in brain tissue including the cerebellum [61]. The PHF12A protein is a component of the BRAF-histone deacetylase co-repressor complex, which mediates transcriptional repression of neuron-specific genes in non-neuronal cells [62]. Multiple translocation breakpoints disrupting *PHF21A* have been reported as causing intellectual disability [63] and alteration of *PHF12A* expression in the cerebellum might contribute to the ataxia seen in this case but we were unable to find any evidence that *PHF21A* could be causing the iris malformation. The breakpoint on the X chromosome disrupts *ARHGAP6*, which is highly expressed in kidney, heart, skeletal muscle, retina and fetal brain. *ARHGAP6* encodes a guanine nucleotide exchange factor that activates Rho-GTPase to regulate signaling interactions within the actin cytoskeleton [64,65]. However, there is no human genetics evidence as yet that mutations in this gene are associated with any developmental disorder. We were also unable to find mutations in the neighbouring gene, *HCCS*, in the other Gillespie syndrome cases in our cohort. *HCCS* has been associated with syndromic microphthalmia [66]. It seems reasonable to consider individual 1371 as having a composite phenotype with *PHF21A*-disrupting breakpoint exacerbating the neurodevelopmental problems but the Gillespie syndrome being, as yet, unexplained.

Perhaps the most significant finding in this study is that we were unable to identify mutations in 27/42 individuals with aniridia and no detectable intragenic mutations in *PAX6*. Although there could be unidentified mechanisms for disrupting PAX6 function, our results also suggests that there may be as yet undiscovered genetic loci responsible for a considerable proportion of aniridia. Whole genome sequence analysis would be an attractive technique for the identification of novel causative mutations in the *PAX6* region, and others involving new loci in *PAX6*-negative individuals with syndromic or isolated aniridia. The high frequency of *cis*-regulatory mutations that we have identified in this cohort highlight the importance of surveying the whole genome. This study has also confirmed that the majority of cases with Gillespie syndrome are not associated with detectable mutations at the *PAX6* locus.

## Supporting Information

S1 Fig Array CGH data for *PAX6* deletion individuals. (DOCX)

S2 Fig Array CGH data for *PAX6* telomeric deletion individuals. (DOCX)

S1 Table Details of the clinical diagnoses of all the patients used in this study, including the genetic pathology (where applicable). (DOCX)

S2 Table Primer sequences and PCR conditions used for amplification and sequencing of the *PAX6*, *FOXC1*, *PITX2*, *PHF21A* and *ARHGAP6* (DOCX)

S3 Table Occurrence of particular descriptive phenotypes in cases with and without a molecular diagnosis. (DOCX)
