# Supplementary figures and images for: Genetic Analysis of ‘PAX6-Negative’ Individuals with Aniridia or Gillespie Syndrome
Source: PLoS One. 2016 Apr 28;11(4):e0153757. doi: 10.1371/journal.pone.0153757 (PMC4849793; doi:10.1371/journal.pone.0153757)

**Genetic Analysis of ‘*PAX6*-negative’ Individuals with Aniridia or Gillespie Syndrome**

**S1 Fig**


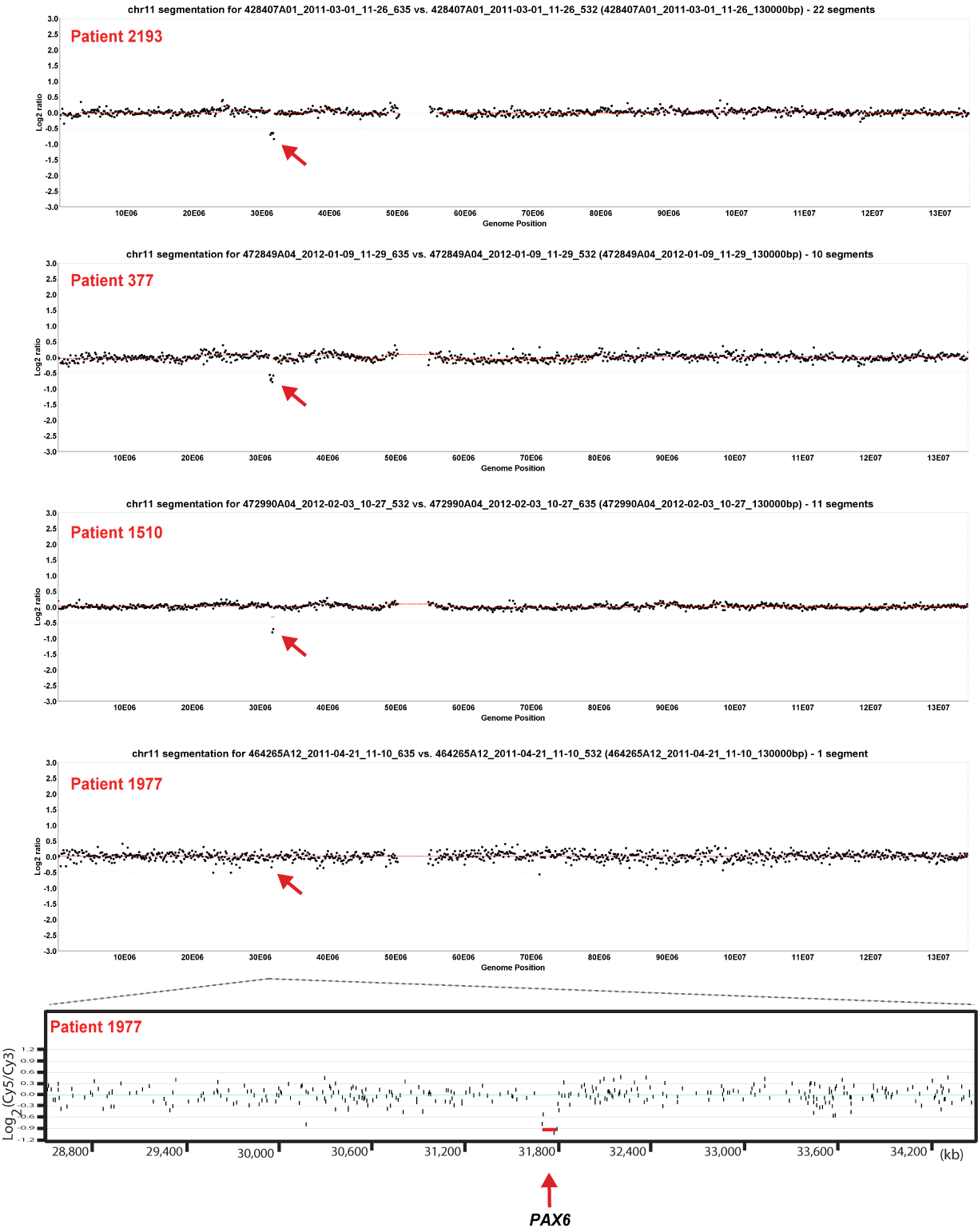

Supplement: S1 Fig — (DOCX) [file pone.0153757.s001.docx]

**Genetic Analysis of ‘*PAX6*-negative’ Individuals with Aniridia or Gillespie Syndrome**

**S2 Fig**

**
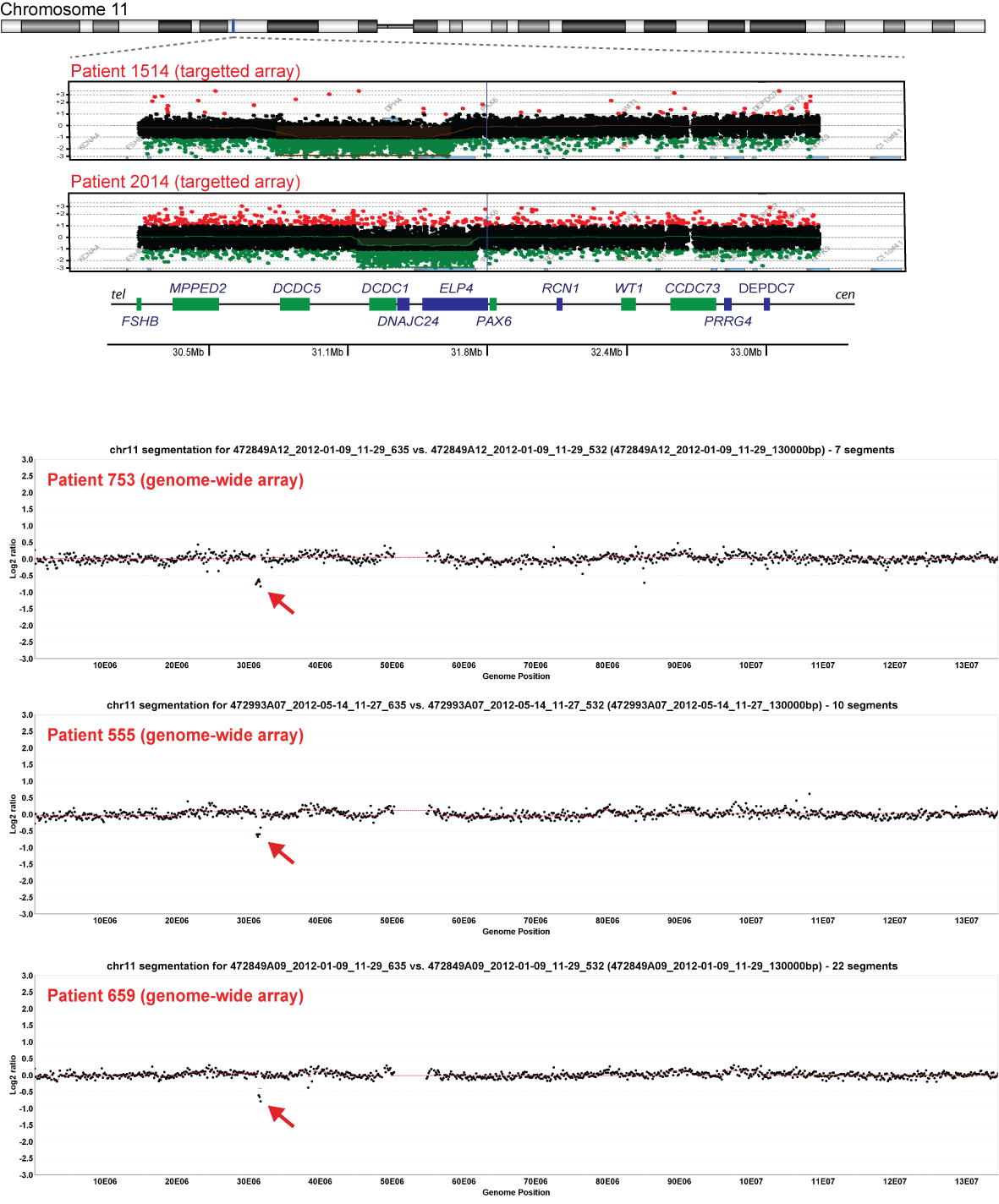
**

Supplement: S2 Fig — (DOCX) [file pone.0153757.s002.docx]
