# Supplementary material for: Genetic Analysis of ‘PAX6-Negative’ Individuals with Aniridia or Gillespie Syndrome
Source: PLoS One. 2016 Apr 28;11(4):e0153757. doi: 10.1371/journal.pone.0153757 (PMC4849793; doi:10.1371/journal.pone.0153757)
Supplement: S1 Table — (DOCX) [file pone.0153757.s003.docx]

**Genetic Analysis of ‘*PAX6*-negative’ Individuals with Aniridia or Gillespie Syndrome**

**S1 Table** - Details of the clinical diagnoses of all the patients used in this study, including the genetic pathology (where applicable).

| **Patient ID** | **Sex** | **Clinical feature** | **Full phenotype description** | **Family history** | **Genetic pathology** | **Confirmation** |
| --- | --- | --- | --- | --- | --- | --- |
| 91 | F | Gillespie | Gillespie syndrome. Late learning to walk and has always had balance problems | No | - |  |
| 182 | M | Aniridia | Partial aniridia, atrophic iris, congenital cataracts, nystagmus, corneal endothelial degeneration. | - | - |  |
| 224 | F | Aniridia | Aniridia | - | - |  |
| 262 | M | Gillespie | Gillespie syndrome, partial hypoplasia of the iris bilaterally. Developmentally delayed with limb & truncal ataxia, no speech yet, slightly delayed myelination | No | - |  |
| 291 | F | Gillespie | Possible Gillespie syndrome, iris hypoplasia. hypotonia, psychomotor retardation & ataxia | No | - |  |
| 377 | F | Aniridia | Bilateral aniridia | - | *PAX6* whole-gene deletion (chr11:31,394,000-31,914,000) |  |
| 404 | M | Aniridia | Unilateral microphthalmia left eye, absent left optic nerve, variant aniridia | - | *-* |  |
| 415 | F | Aniridia | Unilateral aniridia, microphthalmia | - | - |  |
| 535 | F | Aniridia | Bilateral aniridia. Concerns regarding declining head circumference - down to 0.4th centile | No | - |  |
| 555 | F | Aniridia | Bilateral aniridia. VA in right is 6/12 and she has no nystagmus or macular hypoplasia. Left has undergone penetrating graft because of herpes keratitis and VA is poor as graft decompensated so that other parts of the eye cannot be examined. | - | *PAX6* telomeric deletion (chr11:31,108,579-31,649,842) |  |
| 659 | F | Aniridia | Aniridia, possible macular hypoplasia, OD pannus with deep vascularisation of the cornea, corneal dystrophy  Limbal stem cell insufficiency. Nystagmus  Pseudophakia at follow-up | - | *PAX6* telomeric deletion (chr11:31,379,000-31,708,000) |  |
| 660 | F | Aniridia | Anterior and posterior polar cataract. Possible macular hypoplasia | - | - |  |
| 698 | M | Aniridia | Variant aniridia, extensive absence of iris, bad keratopathy, very bad glaucoma, retinal detachment | Yes | - |  |
| 753 | M | Aniridia | Autosomal dominant aniridia | Yes (AD) | *PAX6* telomeric deletion (chr11:30,967,000-31,704,000) |  |
| 1194 | F | Aniridia | Aniridia, secondary glaucomatous severe disease with pressures at 50mmHg on the left and 40mmHg on the right. Very large eyes with 15mm corneal diameter on the right and 19mm on the left. bilateral central corneal opacification and highly cupped optic nerve heads. Right eye is functional while left one has normal pressure but no vision and may need enucleation. . | No | Deletion extending telomeric to *PITX2* (chr4:111,994,000-115,504,000) | qPCR |
| 1234 | M | Aniridia | Variant aniridia variant and optic nerve hypoplasia, severe visual impairment, unusually shaped right pupil, VEP indicates post retinal dysfunction. Plagiocephaly | No | - |  |
| 1246 | F | Aniridia | Peters’ anomaly, bilateral aniridia, buphthalmos, hypertelorism. Had a heart murmer. Heart defect (ASD/PDA) and some dysmorphic features. Noted to have an abdominal wall defect in utero.Looked like an exomphalos at birth but at surgery it was more like a persistent vitellointestinal duct as well as aMeckel’s diverticulum . She had delayed development and facial dysmorphism with marked hypertelorism. | - | *FOXC1* whole-gene deletion (chr6:1,543,591-1,675,085) | TWO MICROARRAY PLATFORMS |
| 1271 | F | Aniridia | Bilateral aniridia. Bilateral Cataract surgery. Normal development | - | - |  |
| 1371 | F | Gillespie-like syndrome | Gillespie-like Syndrome, superior atypical coloboma foveal hypoplasia. Inferior vermis hypoplasia | No | Translocation t(X;11)(p22.32;p12) | FISH |
| 1388 | F | Gillespie | Iris hyploplasia, pyschomotor delay, severe hypotonia, normal MRI without cerebellar hypoplasia, slight facial dysmorphism | No | - |  |
| 1444 | M | Gillespie | Iris hypoplasiaplus agenesis of optic nerves without microphthalmia or microcephaly, Normal MRI at age 2, developed autistic behaviour | No | - |  |
| 1449 | F | Gillespie | Iris hypoplasiawith glaucoma, patent ductus arteriosus, agenesis of the vermis & megacisterna, magna (Dandy Walker anomaly), hypotonia, anal displacement & hypertelorism | No | *FOXC1* whole-gene deletion (chr6:1,543,591-1,675,085) | TWO MICROARRAY PLATFORMS |
| 1497 | F | Aniridia | Right sided microphthalmia/microcornea, ASD, surgery for bilateral congenital cataracts, nystagmus, abnormal iris architecture | Yes (AD) | - |  |
| 1503 | F | Aniridia | Bilateral aniridia with cloudy corneas & glaucoma. Significant developmental delay, growth retardation | - | - |  |
| 1510 | F | Aniridia | Aniridia | - | *PAX6* whole-gene deletion (chr11:31,779,000-31,933,000) |  |
| 1514 | M | Aniridia | Incomplete aniridia | Yes (AD) | *PAX6* telomeric deletion (chr11:30,874,642-31,654,833) | FISH |
| 1634 | F | Aniridia | Bilateral aniridia, corneal clouding, glaucoma | No | *FOXC1* c.302T>C p.(Leu101Pro) *de novo* |  |
| 1635 | M | Aniridia | Congenital anterior chamber dysgenesis associated with nystagmus & poor vision, variant aniridia. Abnormalities include defects in iris inferiorly in each eye, vascularisation of peripheral cornea, inferior lens opacities and foveal hypoplasia | No | - |  |
| 1648 | M | Aniridia | Aniridia. No other features of WAGR Syndrome | - | - |  |
| 1653 | F | Aniridia | Congenital glaucoma, aniridia. Endothelial cell dysfunction | Yes (AD) | - |  |
| 1701 | F | Aniridia | Partial aniridia, anterior chamber malformation of the iris, pupil is very large and iris tissue centrally to the collarette is absent. However there is abundant remains of peripupillary membrane rising from what seems to be the edge of the pupil. Persistent ductus arteriosus & double kidneys | No | - |  |
| 1763 | M | Aniridia | Totally blind, bilateral leucocoria at six weeks caused by bilateral retinal detachments, virtually no iris tissue and ectopic uvea, no suggestion of retinopathy of prematurity and changes were felt to be different from those seen in Norrie’s disease. Development normal no dysmporphic features | No | - |  |
| 1782 | F | Aniridia | Aniridia and corneal clouding | No | - |  |
| 1839 | M | Aniridia | Bilateral aniridia, Peters’ anomaly left eye, right eye with cornea clearing slightly . Thought to be moderately deaf but tests by audiology found to be normal | No | *FOXC1* c.235C>A p.(Pro79Thr) *de novo* |  |
| 1977 | F | Aniridia | Bilateral aniridia, cataract | Yes (AD) | *PAX6* whole-gene deletion (chr11:31,698,271-31,794,414) | FISH |
| 2014 | F | Gillespie | Bilateral iris hypoplasia , Gillespie syndrome. Delayed development, movement disorder with features both ataxia & dystonia, glue ear, some hearing loss requiring tubes | No | *PAX6* telomeric deletion (chr11:31,234,395-31,751,815) | FISH |
| 2018 | F | Gillespie | Genetic ataxia cerebral palsy associated with a developmental abnormality of the iris, Gillespie syndrome | No | - |  |
| 2021 | F | Gillespie | Gillespie syndrome | No | - |  |
| 2193 | F | Aniridia | Aniridia | Yes (AD) | *PAX6* whole-gene deletion (chr11:31,199,000-31,849,000) | FISH |
| 2374 | F | Gillespie | Partial, circumpupillary iris hypoplasia, Gillespie syndrome. Cereballar hypoplasia, psychomotor delay, hypotonia, abnormal speech development | No | - |  |
| 2546 | M | Aniridia | Nystagmus, cataract, aniridia, glaucoma, now totally blind in both eyes | Yes (AD) | - |  |
| 3354 | M | Aniridia | nystagmus since birth, reduced visual acuity to 0.3 (right eye, huge coloboma left eye), macular aplasia, iris transluminancy combined with coloboma of both irides, choroid and retina. | - | - |  |
