# Supplementary material for: Genetic Analysis of ‘PAX6-Negative’ Individuals with Aniridia or Gillespie Syndrome
Source: PLoS One. 2016 Apr 28;11(4):e0153757. doi: 10.1371/journal.pone.0153757 (PMC4849793; doi:10.1371/journal.pone.0153757)
Supplement: S2 Table — (DOCX) [file pone.0153757.s004.docx]

**Genetic Analysis of ‘*PAX6*-negative’ Individuals with Aniridia or Gillespie Syndrome**

**S2 Table** - Primer sequences and PCR conditions used for amplification and sequencing of the *PAX6*, *FOXC1*, *PITX2*, *PHF21A* and *ARHGAP6*

| ***Gene*** | **Exon** | **Forward Oligonucleotide** | **Reverse Oligonucleotide** | **Optimal annealing temperature/cycles** |
| --- | --- | --- | --- | --- |
| ***PAX6*** | **1** | GGGGAGAGGGAGCATCCAAT | TGTCAGCGGCTGGAGAGTGA | 63^0^C/30 cycles |
|  | **2** | GCCGCTGACAGCCCATTTTA | GCGAGAAGAAAGAAGCGGACTC | 62^o^C/30 cycles |
|  | **3** | CCTGATGCAGCTGCCCGAGGAT | CCCGAGCCCGAAGTCCCAGA | 54^o^C/30 cycles |
|  | **4** | AACAGAGCCCCATATTCGAG | AGTCCCTGTGTCCTCCCC | 57^o^C/30 cycles |
|  | **5** | CTCTTCTTCCTCTTCACTCTGC | GAAATGAAGAGAGGGCGTTG | 56^o^C/30 cycles |
|  | **5a-6** | TTTGATGCATCTTCAGGCAG | GACTCCCCACACTTGACTCC | 58^o^C/30 cycles |
|  | **7** | TGGGTGACTGTGTCTTCAGG | AATGGTTGGGAGAGTAGGGG | 58^o^C/30 cycles |
|  | **8** | TTAAGACTACACCAGGCCCC | TGAAGATGTGGCATTTACTTTG | 56^o^C/30 cycles |
|  | **9-10** | TTGATGCACAGTTTGGTCAAC | GTGAGAGTCAGAGCCCGGAG | 58^o^C/30 cycles |
|  | **11** | AAACCTGTTTGCTCCGGG | CAATGAGGTCCGCAGGC | 60^o^C/30 cycles |
|  | **12** | CAGGTTTGCCTCTCTCCTCACA | CAGCCTGCAGAAAGCAGTGG | 62^o^C/30 cycles |
|  | **13** | CCATGTCTGTTTCTCAAAGGG | AAGCTCAACTGTTGTGTCCC | 56^o^C/30 cycles |
|  | **14_1** | CCCCTGGTGTGTCAGTTCCA | GCACATAAACTTCATCTGTTAACAACC | 59^o^C/30 cycles |
|  | **14_2** | GATCAACAAGCTTTGCCACGA | CATGGAAATAAAATTTGTAACACCACA | 59^o^C/30 cycles |
| ***FOXC1*** | **1_1** | TACTCCGTGTCCAGCCCCAACT | GAACTGGTAGATGCCGTTCAG | 58°C/32 cycles |
|  | **1_2** | CCGGACAAGAAGATCACC | TCTTGATGTCCTGGATGC | 58°C/32 cycles |
|  | **1_3** | AAGACCGAGAACGGTACGTG | ATGATGTTGTCCACGCTGAA | 58°C/32 cycles |
|  | **1_4** | TTCAGCGTGGACAACATCAT | GTGACCGGAGGCAGAGAGTA | 58°C/32 cycles |
|  | **1_5** | TACTCTCTGCCTCCGGTCAC | TGCTTTGGGGTTCGATTTAG | 58°C/32 cycles |
| ***PITX2*** | **1*** | GAAACTCCGCGCCTAGAG | GAACGACCACTCCCACCAC | 60°C/32 cycles |
|  | **4** | CATCTGAGCCCTGCTCACTC | GGGCCAGTGCCCTCTTG | 60°C/32 cycles |
|  | **5** | GGGTCTTTGCTCTTTGTCCC | CCAGAGGCGGAGTGTCTAAG | 60°C/32 cycles |
|  | **6** | CTTCCACGGCTTCTGCC | TTCTCTCCTGGTCTACTTGGC | 60°C/32 cycles |
|  | **7_1** | TAGCGTGTGTGTCGGGG | GGACGACATGCTCATGGAC | 60°C/32 cycles |
|  | **7_2** | GCTATTCCTACAACAACTGGGC | ATACGGAGGAGTCGGCG | 60°C/32 cycles |
|  | **7_3** | GGGCTCCAGTCTCAACAGC | ATTCCCAGTCTTTCAAGGGC | 60°C/32 cycles |
| ***PHF21A*** | **4** | GGAGTTTGTTACTTTAAACAGAGGC | GATGGACTTGTTTACTAATCTTAGCC | 60°C/33 cycles |
|  | **5** | TGGTCCAAACCTGATTAGCC | AATACTCAAACCAATTAATATGACACC | 60°C/33 cycles |
|  | **6** | TGCATAGTCTTCCTGCTACTATTC | GCCTCGAGCAGACATATCTTTAG | 60°C/33 cycles |
|  | **7** | GATGTCAGAGCATATTTCTGTGC | TGAGACAGGACCCAGTTCTATATG | 60°C/33 cycles |
|  | **8** | TTTTCTTCCAAGGTTAAGCAGC | AATCCAGCAGGAGCCTAGAAG | 60°C/33 cycles |
|  | **9** | TGATTAACTTACGATGAAAGCTTGTAG | AGAGCCCATGAGGATTTCTG | 57°C/36 cycles |
|  | **10** | GCTTTTATAGGACTTGAGCAGC | CCATATGCCTCAACGACAAG | 60°C/33 cycles |
|  | **11** | TTAAACTTCTGATTGCTTCATCC | TCATTTCATCATTCAGAGAGCAG | 60°C/33 cycles |
|  | **12** | TGGCTTTGTGAGAACCCAG | TCAGGAACAAAGCTGTGGG | 58°C/36 cycles |
|  | **13** | CCTGATTCTGATTTCTTATGGG | TTCAGCAGGTTTTCAGAGGG | 60°C/33 cycles |
|  | **14** | TGGTCTTTAGCTTGTGCCAG | GAGAAGAAACACACACACAAAGC | 60°C/33 cycles |
|  | **15** | CCCTCTCTTTCCCTCCCC | CGCTTTTCCCAATTTAAGCTC | 58°C/36 cycles |
|  | **16** | GAACGAGGCAGCTGATTGTC | CGGACTGGGAGAGAGAAGAG | 60°C/33 cycles |
|  | **17** | CTGCCAGTAGCCACAGATACC | TCCAATAGTTAAGGGCAAAAGG | 58°C/36 cycles |
|  | **18** | GAGCACAGAGGACATTTCAAAG | AAGGCCTAGGTCTCTGCACC | 60°C/33 cycles |
|  | **19** | CTGCCAGGCCTGTTAGAAAC | GCTTTGCTTTTCTAGAGTCTTCAG | 60°C/33 cycles |
| ***ARHGAP6*** | **1_1** | GACCCAAAGCCACCAGC | GATGGTGACTTCTCCTGCG | 58°C/36 cycles |
|  | **1_2** | GTCCTCTTCCCGGGGTC | TAGTGGGTGCCTTGCTCTG | 58°C/36 cycles |
|  | **2** | AAGAGAGGATGAGATTTGTGAGAG | CAGAGCCAATATTTGACTTGC | 58°C/36 cycles |
|  | **3** | GCTTTTGCTCAAATGTAATTATCG | GCAATTCAATAACCCCACC | 58°C/36 cycles |
|  | **4** | CAATTCTTCAGAAAACATTCCTTTG | CACCAACTACATATCCAGTTTTCAC | 56°C/35 cycles |
|  | **5** | TGTATTGGTTAGCATCTGGGG | CAAGCAGAATTGACATTGGG | 56°C/35 cycles |
|  | **6** | TTCCTCCAGGGTCTCAAGC | TTTGCATAAACTTGAACTTTGC | 56°C/35 cycles |
|  | **7** | GCACTGCTCATTGAGGTATGG | GCATAAAGTTATGATGAATGAGTTGG | 59°C/32 cycles |
|  | **8** | GCCTGAATAGTATGGGCTCC | ATGGCCATTTTGGTGGTATG | 60°C/33 cycles |
|  | **9** | TCACCCAGTATCTTGAAAGGC | GAATTTCATCCTGCCTCCAG | 58°C/36 cycles |
|  | **10** | TCTTGCAGCTTTTAGGGCTC | TGCCAAAATTCCACTTCAGG | 58°C/36 cycles |
|  | **11** | GCCATATTGTTTAAATCTGGTCAC | CATGCAACAAGAGGAGGGTC | 58°C/36 cycles |
|  | **12** | CAGGATCTTGAATACAATACTTTGC | CTCCCATTTTCAGCATTTCC | 58°C/36 cycles |
|  | **13_1** | ATTTGAATCCCCAGCAAGG | CGCTTGTCATCCCTCCC | 60°C/36 cycles |
|  | **13_2** | ACGACCTCAGCGAGAGTGAG | CAGTGGCCACCACCCAC | 60°C/36 cycles |

The following cDNA reference sequences were used: *PAX6*, NM_000280.3 (including the alternatively-spliced exon 5a); *FOXC1*, NM_001453; *PITX2*, NM_001204397; *PHF21A*, NM_016621; *ARHGAP6*, NM_013427. Exon numbering is based on the total count, including coding and non-coding (untranslated regions) exons. **PITX2*, NM_000325
