# Supplementary material for: Genetic Analysis of ‘PAX6-Negative’ Individuals with Aniridia or Gillespie Syndrome
Source: PLoS One. 2016 Apr 28;11(4):e0153757. doi: 10.1371/journal.pone.0153757 (PMC4849793; doi:10.1371/journal.pone.0153757)
Supplement: S3 Table — (DOCX) [file pone.0153757.s005.docx]

**Genetic Analysis of ‘*PAX6*-negative’ Individuals with Aniridia or Gillespie Syndrome**

**S3 Table** – Occurrence of particular descriptive phenotypes in cases with and without a molecular diagnosis.

|  | Descriptive term | | | | | |  |
| --- | --- | --- | --- | --- | --- | --- | --- |
|  | Count of partial/variant aniridia | Count of corneal disease | Count of cataract | Count of glaucoma | Count of microphthalmia/coloboma | Count of extraocular disease | Count of Patient ID |
| No mutation detected | 16 | 4 | 5 | 2 | 4 | 10 | 27 |
| Deletion extending telomeric to *PITX2* (chr4:111,994,000-115,504,000) |  |  |  | 1 |  |  | 1 |
| *FOXC1* c.235C>A p.(Pro79Thr) *de novo* |  | 1 |  |  |  | 1 | 1 |
| *FOXC1* c.302T>C p.(Leu101Pro) *de novo* |  | 1 |  | 1 |  |  | 1 |
| *FOXC1* whole-gene deletion (chr6:1,543,591-1,675,085) | 1 |  |  | 2 |  | 1 | 2 |
| *PAX6* telomeric deletion (chr11:30,874,642-31,654,833) | 1 |  |  |  |  |  | 1 |
| *PAX6* telomeric deletion (chr11:30,967,000-31,704,000) |  |  |  |  |  |  | 1 |
| *PAX6* telomeric deletion (chr11:31,108,579-31,649,842) |  | 1 |  |  |  |  | 1 |
| *PAX6* telomeric deletion (chr11:31,234,395-31,751,815) | 1 |  |  |  |  | 1 | 1 |
| *PAX6* telomeric deletion (chr11:31,379,000-31,708,000) |  | 1 |  |  |  |  | 1 |
| *PAX6* whole-gene deletion (chr11:31,199,000-31,849,000) |  |  |  |  |  |  | 1 |
| *PAX6* whole-gene deletion (chr11:31,394,000-31,914,000) |  |  |  |  |  |  | 1 |
| *PAX6* whole-gene deletion (chr11:31,698,271-31,794,414) |  |  | 1 |  |  |  | 1 |
| *PAX6* whole-gene deletion (chr11:31,779,000-31,933,000) |  |  |  |  |  |  | 1 |
| Translocation t(X;11)(p22.2;p11.2) | 1 |  |  |  | 1 | 1 | 1 |
| **Grand Total** | **20** | **8** | **6** | **6** | **5** | **14** | **42** |
